# Case of a Gentleman Who Was Born Deaf and Dumb, and Subsequently Became Insane

**Published:** 1855-10-01

**Authors:** James B. Balfour

**Affiliations:** Edinburgh.


### CASE OF A GENTLEMAN WHO WAS BORN DEAF AND DUMB, AND SUBSEQUENTLY BECAME INSANE.

BY JAMES B. BALFOUR, M.D., EDINBURGH.

No apology is necessary, on my part, in presenting the following case to the notice of my professional brethren, as I believe it is one which is perfectly unique. The only apology necessary is, that such an interesting case should not have been related by one far more able to elucidate the many scientific points which this presents, both to the medical man and the psychologist. Perhaps some excuse may be found in the fact, that it came under my observation at an early period of my professional studies, and before I was able to bring that knowledge, subsequently acquired, to bear on a subject of such deep importance. I give the case as I find it in the notes, which I at the time made, with the remarks which I then appended to it. And I will certainly feel glad if any of your contributors, engaged more immediately with the study of psychological science, will favour us at some future time with their remarks upon it.

The subject of my memoir was a native of Ireland, belonging to a wealthy and highly respectable family. He was born deaf, and, as might be expected, never acquired the power of imitating sounds, or, in other words, was dumb. When he grew up, his friends sent him to Edinburgh, to be under the care and teaching of the famous Mr. Kinniburgh. While at this school he made great proficiency in learning, and exhibited a more than usual amount of talent. He remained some years with Mr. Kinniburgh, and when his powers were considered sufficiently developed, he was removed by his friends to his native country, in one of the flourishing commercial towns of which, through the influence of a relative, he obtained a situation as a clerk. While he held this situation he displayed great proficiency in book-keeping, and the general transactions of business; and so high did he rise in the estimation of his employers, not only from his general aptitude for business, but also from his quiet, amiable manners, that he was their head-clerk, which situation he retained for many years, and, indeed, until he became incapable of doing so from mental aberration. From a child he was remarkable for the healthiness of his constitution, but, as he grew up, he gradually became very stout and corpulent, and when about thirty-five years of age, he was suddenly attacked by a fit of apoplexy. From this he recovered, but ever afterwards he was observed to have a slight dragging of the right leg, showing that there was some permanent lesion of the brain. It was after this attack that the first signs of mental aberration began to present themselves; he was not so correct in his book-keeping, and it was discovered, when holding communication with him, that he entertained some strange notions in regard to religion, which was the more remarkable, as previous to this period he had been very strict in his religious tenets, and a member of the Protestant church. The ideas which he now began to hold, as might be easily anticipated, could never be very accurately ascertained, but they appeared to have reference to some mysterious connexion which existed between himself and the Saviour; and other ideas of a similar nature. His conduct, at the same time, began to be influenced by the ideas he entertained, and it was ultimately found necessary to remove him to a place of confinement. He was, therefore, removed to an asylum for the insane, and it was while there that I had an opportunity of observing him.

The dragging of the right leg still continued to a slight extent, but otherwise, he was in robust health. He was merry and good-natured, and soon became quite happy in his new situation. No clear idea could ever be obtained of the state of his mind, so far as his delusions were concerned, as he always avoided entering upon those topics on which he was said to be insane (a peculiarity this, not confined to a case like his, but very common amongst insane persons). He had at this time lost, to a great extent, the use of the finger alphabet, but he held communication with those around by means of writing. He always carried his slate with him, and it afforded him great pleasure when any one would hold communication with him by its means. He was a great reader, and always selected his own books. He asked for the library catalogue, and pointed out the book or books he wished to peruse, and it was remarkable how he selected books of interest, both in history and miscellaneous literature. He appeared to enjoy such passages in the works which he read, as exhibited wit and humour, for he was often observed laughing to himself while reading, and on being interrogated what made him do so, he pointed to the passage, and it was invariably found to be one which would excite the risibility of a sane person. There was one peculiarity about him which was curious, and which is particularly worthy of notice, as there can be no doubt it was connected with his insanity—it must have had some connexion with his delusions, could we have discovered it. He believed he was not deaf and dumb, but that he could speak perfectly well, and it was those around him who were in that condition. He would write this down upon his slate, and when asked to give a specimen of his talkative powers, would commence to utter the most discordant sounds, and if told that was not talking at all, he would get rather ill pleased, and state that it arose entirely from the stupidity and deafness of the party inquiring, who could not understand what he said. All this conversation, remember, all the while, being carried on by writing on his slate.

During his residence in the asylum, he mingled in all the amusements which are continually had recourse to in such institutions of the present day, in order to relieve the patients of the ennui of confinement, and to endeavour to rouse their dormant faculties, or drive away their morbid thoughts. He attended the musical concerts, and although the sweet sounds of the music, in his case fell upon ears closed to all their beauty, he yet appeared pleased with the exhibition, and clapped his hands and laughed when others did it. And at the little select evening re-unions which frequently took place, he was the happiest of all.

I now come to give an account of one of the most interesting scenes it has ever been my fortune to witness. In the neighbouring town to where the asylum is situated, there is a school for deaf and dumb children, which is taught by a gentleman who is also deaf and dumb. Upon one occasion the children were all brought to the asylum to exhibit before a number of the patients their proficiency in learning. Great interest was exhibited in this exhibition by the patients present; and the patient who is the subject of this notice was present among the rest, and his enjoyment appeared unbounded. After the exhibition was over, he was introduced to the teacher of the children, and they began to converse together. The teacher told him he was educated at Kinniburgh's, and asked where he had been taught. On hearing this the patient looked intently at him for some moments, suddenly uttered a cry of joy, and rushed into his arms. The expression of his joy is such as we could fancy being made by a wild Indian. He danced, laughed, and screamed in turns; and it was some time ere those around could understand the cause of such unusual excitement. At length the teacher explained that the two had been intimate acquaintances at Kinniburgh's, eighteen years before; and although thus long separated, and never having met during the interval, he had recognised in the person before him his old school companion. Memory had recalled the happy days of his youth, and his joy burst forth in the manner I have described. It has never been my lot to witness a more interesting meeting, and it would require a far abler pen than mine to convey even a faint idea of the scene which took place. The joy he exhibited upon this occasion was the more remarkable, as he was from time to time visited by a favourite brother, and although he always appeared glad to see him, yet he never made any marked demonstration of joy. After this the teacher frequently visited him, and he always exhibited marked pleasure at the meetings, both by signs and noises. About this time, however, he had another apoplectic attack, from which he recovered so far as to be able to be out of bed, but he was now a changed man; his power of voluntary motion was greatly impaired, and instead of being merry and active, he spent the greater part of his time in a state of sleep. He could not be induced to read or converse by means of writing. At times, indeed, a faint glimmering of his former cheerfulness would return, but only as it were to show that the mind was still there, and he would again relapse into his condition of drowsiness. The mind was, as it were, locked up, and even the ordinary means of access under such circumstances were denied to us. He continued thus for two or three months, dead to the external world, and leading almost an animal existence, when a third attack of apoplexy put a period to his sufferings, cares, and trials.

In this case, a *post mortem* examination was kindly permitted to be made; and I noted the following as the appearances presented. I can only regret, that some one more capable than I am, had not been present to give me the aid of his powers of examination and observation.

As might have been expected, there was no lesion observable of either the abdominal or thoracic viscera. On opening the head, the blood-vessels on the surface of the brain were found to be more than usually congested; the brain itself was found to be large and firm, and on cutting into its substance, presented a congested appearance. There was slight effusion into both ventricles. There was marked softening of the left corpus striatum, though not to a great extent. Both internal ears were removed, and in company with a friend, who was distinguished for his anatomical knowledge, I examined them most minutely, but we could discover nothing either in their structure or arrangement, which could account for the deafness; everything appeared quite natural. We had no microscope at our command to examine the state of the nerve, otherwise this would have been done, but, so far as ocular demonstration went, it also appeared to be natural.

Having thus brought the history of this extraordinary case to a conclusion, I would only add one or two remarks which suggest themselves. In the first place, from what cause did the deafness arise? We have seen, from the *post mortem* appearances, that it could not have arisen from any malformation of the ear itself, as all parts of that organ were perfectly natural. Did it then arise from some defect of the auditory nerve, incapacitating it from conveying impressions made upon the ear to the sensorium? Or did it arise from some affection of the sensorium itself in connexion with the auditory nerve? I have already said that I was not able, microscopically, to examine the nerve and brain, and, therefore, cannot say whether any organic change existed. We are still very ignorant of what are the causes of many cases of congenital deafness, and it is a subject which offers great scope for microscopical investigation, as in many cases there is nothing in the structure of the ear, or in the auditory nerve itself, which, to the naked eye, can account for this infirmity.

Another interesting point is, the cause of the extraordinary mental phenomena presented by this patient. This case presents one of a most interesting character for the study of the psychologist, and I trust some of my brethren in the profession will take up this point, and present us with their views upon the subject.

I have given the case as fully as possible, from the notes I made at the time; and I trust I will be excused for not entering more minutely than I have done into the points of interest which it presents.—*Edinburgh Medical Journal.*

---

Amongst the prevalent *on dits* is one relative to a vacancy on the Medical Board of Visiting Commissioners in Lunacy, which it is said will immediately occur. It is well known the Lord Chancellor has this piece of patronage in his gift. We have no doubt that his lordship will weigh the special psychological qualifications of each candidate (for we understand there are many in the field), and will only select for the vacant post a gentleman who has established, by his labours in reference to the subject of insanity, the treatment of the insane, and the management and organization of Lunatic Asylums, that he is in all right essentials fully qualified to perform the onerous duties of so responsible an appointment to the satisfaction of the profession, the public, and all who take an interest in the amelioration of the condition of the insane.

